# New Insights on the Role of Lipid Metabolism in the Metabolic Reprogramming of Macrophages

**DOI:** 10.3389/fimmu.2019.02993

**Published:** 2020-01-10

**Authors:** Ana Batista-Gonzalez, Roberto Vidal, Alfredo Criollo, Leandro J. Carreño

**Affiliations:** ^1^Facultad de Odontología, Instituto de Investigación de Ciencias Odontológicas, Universidad de Chile, Santiago, Chile; ^2^Facultad de Ciencias Químicas y Farmacéuticas and Facultad de Medicina, Advanced Center for Chronic Diseases, Universidad de Chile, Santiago, Chile; ^3^Millennium Institute on Immunology and Immunotherapy, Universidad de Chile, Santiago, Chile; ^4^Programa de Microbiología y Micología, Instituto de Ciencias Biomédicas, Facultad de Medicina, Universidad de Chile, Santiago, Chile; ^5^Programa de Inmunología, Instituto de Ciencias Biomédicas, Facultad de Medicina, Universidad de Chile, Santiago, Chile

**Keywords:** macrophages, lipid metabolism, metabolic reprogramming, oxidative phosphorylation, fatty acid oxidation

## Abstract

Macrophage activation is intimately linked to metabolic reprogramming. Inflammatory (M1) macrophages are able to sustain inflammatory responses and to kill pathogens, mostly by relying on aerobic glycolysis and fatty acid biosynthesis. Glycolysis is a fast way of producing ATP, and fatty acids serve as precursors for the synthesis of inflammatory mediators. On the opposite side, anti-inflammatory (M2) macrophages mediate the resolution of inflammation and tissue repair, switching their metabolism to fatty acid oxidation and oxidative phosphorylation. Over the years, this classical view has been challenged by recent discoveries pointing to a more complex metabolic network during macrophage activation. Lipid metabolism plays a critical role in the activation of both M1 and M2 macrophages. Recent evidence shows that fatty acid oxidation is also essential for inflammasome activation in M1 macrophages, and glycolysis is now known to fuel fatty acid oxidation in M2 macrophages. Ultimately, targeting lipid metabolism in macrophages can improve the outcome of metabolic diseases. Here, we review the main aspects of macrophage immunometabolism from the perspective of the metabolism of lipids. Building a reliable metabolic network during macrophage activation will bring us closer to targeting macrophages for improving human health.

## Introduction

Macrophages are a heterogeneous population of immune cells found in all tissues of the organism (1, 2). They were first discovered and described as phagocytic cells by the Russian zoologist Élie Metchnikoff. Since then, macrophages have emerged not only as mediators of the first line of immune defense but also as key players in tissue homeostasis, development, and pathology (3). One of the main characteristics of macrophages is their plasticity, as they respond to different stimuli by rapidly changing their functional profile in a process called polarization. Classically activated macrophages (M1) are induced by lipopolysaccharide (LPS), toll-like receptors (TLR) ligands, or interferon-gamma (4). Their function is to kill pathogens and to present antigens to T cells to initiate the immune response, and they do so by secreting proinflammatory cytokines such as tumor necrosis factor-alpha, interleukin (IL) 1β (IL1β), IL6, IL12, and IL23 (4). M1 macrophages also express high levels of inducible nitric oxide synthase (4, 5). This enzyme plays an essential role in pathogen killing using arginine to synthesize nitric oxide (NO), which can form reactive oxygen species (ROS) with microbicidal properties (6). Alternatively activated macrophages (M2), on the other hand, are induced by products secreted by innate and adaptive immune cells as a result of parasitic infections, such as IL4 and IL13 (7, 8). These anti-inflammatory macrophages act mainly to resolve inflammation and in tissue remodeling through the secretion of insulin-like growth factor 1 (9), transforming growth factor-beta, and vascular endothelial growth factor (10). In contrast to M1, in M2 macrophages, arginase 1 is induced, and thus, arginine is used to produce polyamines precursors for collagen synthesis, used for tissue repair (11). In addition, different stimuli can induce different subsets of M2 macrophages, such as M2b (activated by TLR ligands or by IL1R agonists), M2c (induced by glucocorticoids or IL10), and M2d (induced by TLR ligands and A2 adenosine receptor agonists) (12).

The notion that M1 and M2 macrophages also differ in their metabolism has been around for more than a decade now, and metabolic adaptations of each type of macrophage intimately respond to their primary function. M1 macrophages are known to rely on aerobic glycolysis and to have impaired oxidative phosphorylation (OXPHOS). This metabolic adaptation favors rapid ATP production to sustain their phagocytic function and provides metabolic precursors to feed the pentose phosphate pathway. In these macrophages, the tricarboxylic acid (TCA) cycle is broken into two parts to provide precursors needed for the synthesis of several lipids (3) and the stabilization of transcription factors such as hypoxia inducible factor 1α, a key player in the activation of glycolysis (13). On the contrary, M2 macrophages have an intact TCA cycle and enhanced fatty acid oxidation (FAO) and OXPHOS (3). Although it was initially thought that proinflammatory macrophages were solely glycolytic and that FAO and OXPHOS were characteristic of anti-inflammatory macrophages, through the years, it has become clear that this equation is not that simple, and recent findings support the need of glycolysis for M2 macrophages, and FAO has been found to occur also in M1 macrophages (14, 15). Here, we present a summary of how lipid metabolism is differentially regulated in M1 and M2 macrophages to promote unique activation and cell functions. Controversial findings are discussed, as well as the potential of using lipid metabolism in macrophages as a target for the treatment of metabolic diseases. Finally, the M1/M2 classification serves us to simplify the rather complex phenomena of macrophage polarization.

## Lipid Biosynthesis and the Inflammatory Response of Macrophages

**Figure 1:**
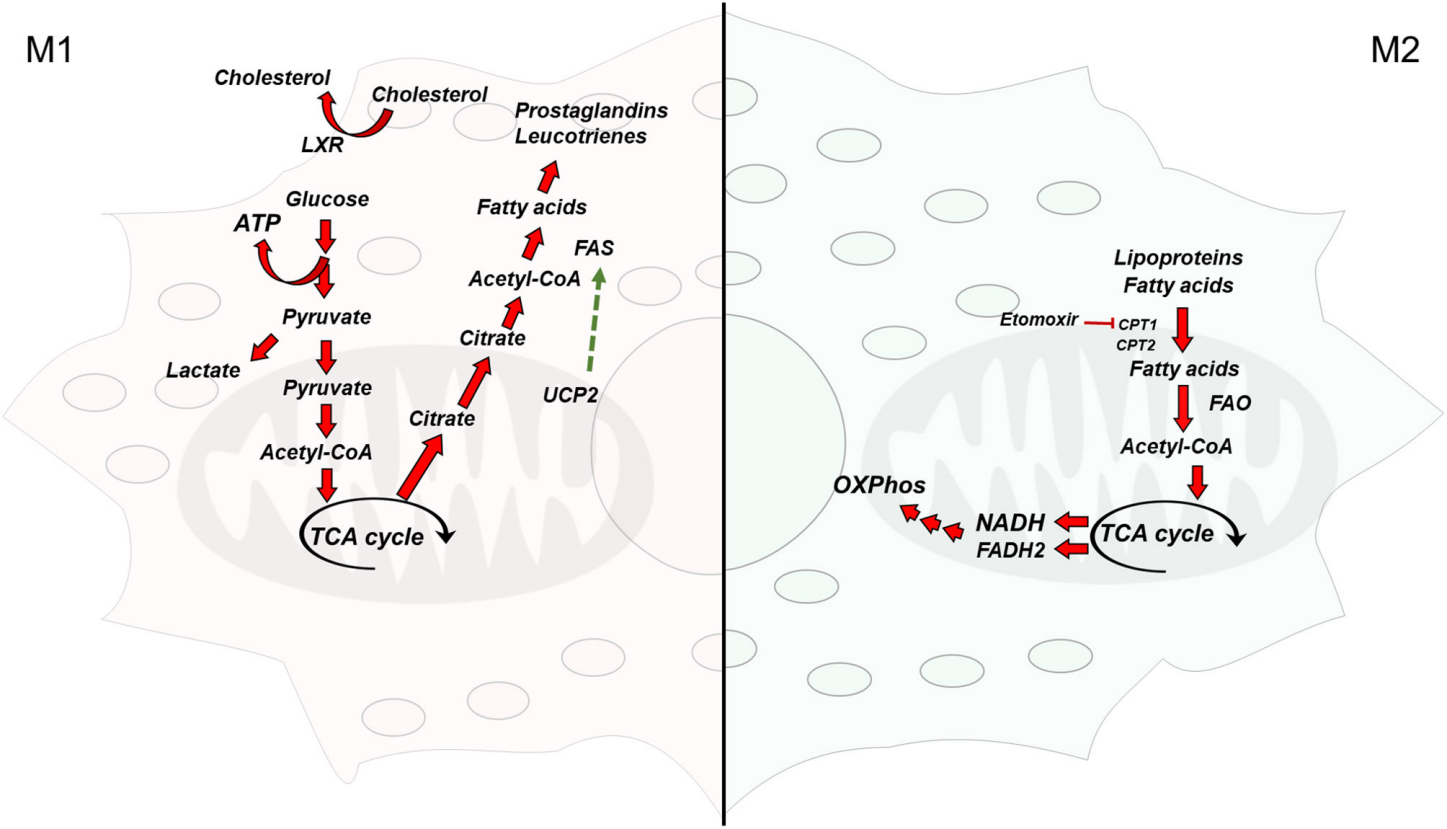
Overview of the metabolic pathways differentially activated in macrophages. **(Left)** Classically activated macrophages are glycolytic and synthesize fatty acids from acetyl-CoA to obtain inflammatory mediators **(Right)** Alternatively activated macrophages rely on fatty acid oxidation and have a functional electron transport chain and oxidative phosphorylation. TCA cycle, tricarboxylic acid cycle; FAS, fatty acid synthase; acetyl-CoA, acetyl-coenzyme A; ATP, adenosine triphosphate; FAO, fatty acid oxidation; NADH, nicotinamide adenine dinucleotide (reduced form); FADH_2_, flavin adenine dinucleotide (reduced form); UCP2, uncoupling protein 2; LXR, liver X receptor; CPT1/2, carnitine palmitoyltransferase 1/2.

Lipogenesis comprehends a series of enzymatic reactions where fatty acids and triglycerides are synthesized. Acetyl-Coenzyme A (acetyl-CoA) serves as a building block for the synthesis of cholesterol, isoprenoids, and fatty acids (16). At the same time, fatty acids are used for the synthesis of triglycerides and complex lipids (16). Lipid biosynthesis is essential for membrane remodeling and the synthesis of inflammatory mediators in M1 macrophages. In these cells, glycolysis is upregulated not only to provide ATP in a faster way, but to fuel the TCA cycle to obtain acetyl-CoA from citrate (Figure 1). In agreement with this, the levels of ATP-citrate lyase (ACLY), the enzyme that converts citrate in acetyl-CoA quickly increases in activated macrophages, and ACLY silencing or inhibition is sufficient to reduce the expression of inflammatory mediators such as NO and ROS (17). Lipogenesis is regulated at the transcriptional level by the sterol regulatory element-binding proteins (SREBPs), which are key elements in the synthesis of fatty acids and cholesterol (18). *Srebp1-a*, one of the three SREBP isoforms, is abundantly expressed in macrophages and positively regulates their inflammatory response. Im et al. showed that LPS induces *Srebp1-a* expression in macrophages and that mice with *Srebp1-a* deficiency have a defective innate immune response (19). Interestingly, macrophages isolated from these mice not only were unable to induce lipid biosynthesis in response to LPS but also secreted lower cytokine levels due to a defective inflammasome. Lipidomic studies have shed light on the importance of lipid metabolism for macrophage polarization toward an inflammatory phenotype. For instance, it is now known that, upon selective TLR4 stimulation, remodeling of glycerolipids, glycerophospholipids, and prenols happens in macrophages (20), which is accompanied by an increase in the synthesis of eicosanoid, sphingolipids, and sterols. Mechanistically, this happens through the activation of the signal transducer and activator of transcription 3 (STAT3) through nuclear factor kappa light-chain enhancer of activated B cells (21).

Another critical player regulating lipid biosynthesis in M1 macrophages is fatty acid synthase (FAS), a key enzyme for fatty acid biosynthesis (22). FAS has proven to be essential for M1 induction. Wei et al. showed that FAS deletion in macrophages prevented adipose macrophage recruitment and inflammation in mice (23). In addition, these mice were resistant to diet-induced insulin insensitivity. The authors showed that FAS is necessary for membrane remodeling in macrophages and that deficiency in FAS led to changes in the composition of the plasma membrane and Rho GTPase trafficking, which blunted the inflammatory signaling in macrophages. The activation of FAS by the mitochondrial uncoupling protein 2 in macrophages also mediates the induction of the NLR family pyrin domain containing 3 (NLRP3) inflammasome and the consequent secretion of IL1β and IL18 in response to an LPS challenge (24). Consistent with this, uncoupling protein 2 deficiency improved survival in a mouse model of polymicrobial sepsis, which was associated with a decrease in FAS-mediated free fatty acid synthesis. In addition to the *de novo* synthesis of fatty acids, the NLRP3 inflammasome can also be activated in macrophages by exogenous lipids, such as palmitate (25), a saturated fatty acid. An increase in the levels of circulating saturated fatty acids occurs during obesity, which is thought to trigger inflammation in adipose tissue in part by activating macrophages inflammasome (15). However, the mechanisms by which endogenous and exogenous fatty acids induce inflammasome activation in macrophages are not fully understood. Recently, Gianfrancesco et al. showed that saturated but not unsaturated fatty acids induced inflammasome activation by increasing the level of saturated phosphatidylcholine. This led to a loss in membrane fluidity and the consequent disruption of the Na^+^/K^+^ ATPase, which caused a K^+^ efflux (25). In addition, Wen et al. showed that palmitic acid induction of inflammasome activation in macrophages requires LPS (26). In summary, in M1 macrophages, lipids serve as precursors for the synthesis of inflammatory molecules, as well as to potentiate inflammasome activation.

## Fatty Acid Oxidation Fuels the Anti-inflammatory Function of M2 Macrophages

In sharp contrast to M1 macrophages, M2 macrophages are characterized by an intact TCA cycle and an enhanced mitochondrial OXPHOS that sustain constant energy production. OXPHOS in M2 macrophages is fueled by fatty acid uptake (27), which are oxidized via FAO (Figure 1). Fatty acid uptake happens through lipolysis of circulating lipoproteins (28) and fatty acids, which are internalized through CD36 (29). In a few cases, lipoproteins are internalized by endocytosis (30, 31). In this sense, IL4 treatment induces triglyceride lipolysis in macrophages (29). The importance of FAO for M2 polarization is highlighted in several studies. Using etomoxir, an inhibitor of the mitochondrial carnitine palmitoyl-transferase 1, Malandrino et al. showed that blocking FAO inhibits the activation of M2 macrophages induced by IL4. In the same note, the expression of a constitutively active carnitine palmitoyl-transferase 1 in cultured macrophages prevented the palmitic acid induction of a proinflammatory phenotype (32). Mechanistically, the activation of the oxidative program in M2 macrophages happens through the activation of the peroxisome proliferator activated receptor-γ (PPARγ) (33) and the proliferator-activated receptor-coactivator 1β (34). PPARγ can sense fatty acids, and it has been shown to mediate the transcription of M2 signature genes upon oleic acid and IL4 stimulation (35). Interestingly, PPARγ was recently found to mediate M2 polarization by promoting the oxidation of glutamine (36), an amino acid that fuels OXPHOS (37). In spite of these advances, it is still not fully understood how FAO and OXPHOS are mechanistically linked to the anti-inflammatory phenotype of M2 macrophages.

## Dual Role of Lipid Metabolism in Macrophage Polarization

Although the consensus is that aerobic glycolysis defines M1 macrophages, and FAO is characteristic of M2 macrophages, several pieces of evidence challenge this paradigm and open questions about the metabolic programming during macrophage activation. For instance, it has been shown that FAO is necessary for inflammasome activation in bone-marrow-derived macrophages (BMDM). Pharmacological and genetic inhibition of NADPH oxidase 4, an enzyme that induces inflammasome activation through CPT1-mediated FAO, inhibits NLRP3 activation, and the consequent secretion of IL1b and IL18 (38). Other studies show that FAO is required for palmitate-induced NLRP3 inflammasome activation, which happens through the mitochondrial oxidation of palmitate and the generation of ROS (26, 39). The notion that M2 macrophages are completely independent of glycolysis has also been questioned. Huang et al. showed that a source of external fatty acids is dispensable for M2 activation as long as glucose is present in the media (29). In agreement with this, the same authors showed that inhibiting glycolysis in IL4-stimulated BMDM blunted the expression of M2 activation markers (14). The authors suggested that glycolysis in M2 macrophages fuels the TCA cycle for fatty acid biosynthesis through FAS, which are used later for FAO. This was further supported by Wang et al. by showing that 2-deoxyglucose, an inhibitor of glycolysis that also impairs OXPHOS indirectly, blunted M2 macrophage activation. However, galactose, a glucose analog that inhibits glycolysis but not OXPHOS, did not affect M2 polarization (40). These data reinforce the idea that in M2 macrophages the function of glycolysis could be to fuel OXPHOS, and inhibiting glycolysis will not affect M2 polarization unless OXPHOS is affected.

Liver X receptors (LXRs) are a family of nuclear receptors that act as cholesterol sensors to control cholesterol efflux and lipogenesis (41, 42). The role of LXRs in macrophage polarization seems to be complex. For instance, it has been shown that LXR-deficient mice are more susceptible to infections with the intracellular bacteria *Listeria monocytogenes* (43) and *Mycobacterium tuberculosis* (44). However, the LXR agonist GW3965, which targets both LRXα and LXRβ, is able to inhibit the LPS-induced expression of inflammatory genes such as inducible nitric oxide synthase, cyclooxygenase 2, and IL6 in macrophages (45). In addition, Bruhn et al. showed that LXR deficiency in mice confers protection against *Leishmani*a infection (46). Furthermore, expressing a constitutively active LXR in macrophages *in vivo* not only drives the synthesis of genes involved in cholesterol efflux but also inhibits LPS-induced expression of inflammatory genes (47).

Much controversy also exists on whether FAO is obligatory for macrophage polarization into the M2 phenotype. Although it was initially demonstrated that FAO is essential for M2 polarization in murine macrophages (14, 29, 32), later, several authors used the CPT1 inhibitor etomoxir to demonstrate that FAO is dispensable for IL4-induced M2 polarization in mouse and human macrophages (48, 49). However, proteomic data revealed that human macrophages seem not to depend on FAO for M2 polarization, but they instead use gluconeogenesis as an energy source (50). This piece of data could explain why in human macrophages M2 polarization is unaffected when FAO is inhibited. More recently, Nomura et al. used a genetic approach to directly assess the role of FAO in macrophage polarization. By deleting *Cpt2* specifically in the myeloid lineage of mice, the authors demonstrated that BMDM lacking *Cpt2* were unable to oxidase fatty acids, as expected. However, they did not find differences in IL4-induced expression of M2 markers between control and *Cpt2-*deficient BMDM (51). Interestingly, the authors also found that etomoxir was able to inhibit M2 polarization in both control and *Cpt2-*deficient BMDM, which suggests that the data based on the use of etomoxir must be taken with caution, since it is possible that CPT1 has other functions outside FAO or that etomoxir is not specific for CPT1 (51). Divakaruni et al. further supported this by showing that etomoxir has off-target effects indeed and that the inhibition of M2 polarization by this compound happens through the alteration of CoA levels rather than the inhibition of fatty acid oxidation (52). Overall, the available data suggest that the role of FAO in macrophage polarization is more complicated than initially thought. Future experiments using genetic approaches rather than pharmacological inhibitors, which have the drawback of potentially being unspecific, will help elucidate not only the role of FAO in macrophage polarization but the molecular mechanism behind this process.

## Targeting Lipid Metabolism to Redirect Macrophage Activation in Metabolic Diseases

Macrophages infiltrate healthy tissue, where they play a critical role in maintaining tissue homeostasis during aseptic conditions (53). Under normal circumstances, M2 polarized macrophages are thought to constitute the majority of macrophages in adipose tissue and are necessary for maintaining insulin sensitivity through the secretion of IL10 (54). However, infiltration of inflammatory macrophages in adipose tissue happens during obesity (55). In addition, an increase in circulating saturated fatty acids is known to activate TLR4 in adipocytes (56). Fatty acid-binding protein 1, an intracellular protein that mediates the uptake of fatty acids, is important in mediating macrophage switch between inflammatory and anti-inflammatory and has been proposed as an interesting target to limit adipose tissue inflammation during obesity (57). Boutens et al. studied the metabolic programming of adipose tissue-resident macrophages in lean and obese mice using transcriptomic analysis and found that adipose tissue macrophages in obese mice have an increase in glycolysis and OXPHOS (58). Interestingly, cytokine release in these macrophages not only depends on glycolysis but also fatty acid oxidation. Future approaches to control adipose tissue inflammation in obese individuals might include the reprogramming of adipose tissue-resident macrophages into an anti-inflammatory phenotype.

Atherosclerosis is an inflammatory disease where cholesterol-rich lipoproteins accumulate in arterial walls leading to their oxidation and the consequent recruitment of several subtypes of immune cells (59). Macrophages are known to internalize oxidized low-density lipoproteins (oxLDL) and lipids within atherosclerotic plaques, which leads to the formation of foam cells with an inflammatory phenotype, all of which contributes to artery occlusion. In addition, lipid uptake causes an increase in ROS in macrophages, which leads to mitochondrial dysfunction and an impaired OXPHOS. This is thought to prevent polarization into the M2 phenotype and to contribute to chronic inflammation and atherosclerosis progress (60). Several authors have shown that targeting lipid metabolism in macrophages can improve atherosclerosis outcomes in mice models. The overexpression of LXRα in macrophages in a mouse model of atherosclerosis had an antiatherogenic effect by increasing cholesterol efflux in macrophages (61). The same effect is seeing using LXR agonists in mice (62). Recently, it was shown that individuals with a specific variant of Perilipin-2, a lipid droplet-associated protein, are less susceptible to the development of atherosclerosis (63). Interestingly, the mechanism behind this protection involves the upregulation of LXR in primary monocyte-derived macrophages from these individuals. In recent years, trained innate immunity has also emerged as a potential mechanism of atherogenesis (64). It is known that macrophages exposed to oxLDL have an enhanced response to restimulation with TLR ligands and have an increased propensity to form foam cells (65, 66). In addition, during the induction of trained immunity, there is an activation of the cholesterol synthesis pathway in macrophages (67).

CCAAT enhancer-binding proteins (C/EBP) are a family of transcription factors involved in adipocyte differentiation with key roles during macrophage polarization (68). In general, it has been proposed that C/EBPα promotes M1 polarization, whereas C/EBPβ participates in M2 activation (69, 70), although several studies suggest that this distinction is more complicated (71, 72). Interestingly, a recent study showed that C/EBPβ was enriched in open chromatin regions in primary human macrophages exposed to oxLDL (73), establishing a link between this transcription factor and the development of atherosclerosis. Overall, redirecting macrophage polarization toward an anti-inflammatory phenotype could have a significant impact on the treatment of metabolic diseases where inflammation plays critical roles.

## Concluding Remarks

Lipids are critical metabolites during macrophage polarization. M1 macrophages synthesize fatty acids to use them as precursors for the synthesis of inflammatory mediators while at the same time obtaining most of the ATP from aerobic glycolysis. This adaptation is most likely a result of the rapid activation of M1 macrophages during inflammatory responses. M2 macrophages, on the other hand, are involved in the resolution of inflammation and thus do not need to produce energy in a fast way. As a consequence, M2 macrophages have a functional mitochondrial respiratory chain fueled by the oxidation of fatty acids. Despite these advances, many questions remain on the role of lipid metabolism for macrophage polarization. During the past decade, several pieces of data have questioned the classical view that M1 macrophages rely only on aerobic glycolysis and M2 depend solely on FAO. The evidence suggests that the metabolism of macrophages during activation is more complex, and more studies are needed to unravel the metabolic signature of macrophages. Furthermore, this task gets complicated by the fact that most of the data on macrophage polarization come from murine studies. However, several differences exist between human and murine macrophages in terms of gene expression (74) and the metabolic pathways activated during polarization (48, 50, 75, 76). This makes difficult the extrapolation from mouse to humans, especially whether the reprogramming of macrophage polarization by metabolic interventions will be helpful in the treatment of human diseases. More importantly, the question of whether metabolic adaptations are the cause or the consequence of macrophage polarization needs to be addressed. Answering these questions will take us one step further to understand the relation between macrophage activation and metabolism.

## Author Contributions

AB-G and LC wrote the article and made figures. AB-G, RV, AC, and LC reviewed the article.

### Conflict of Interest

The authors declare that the research was conducted in the absence of any commercial or financial relationships that could be construed as a potential conflict of interest.
